# Simultaneous assessment of mitochondrial DNA copy number and nuclear epigenetic age towards predictive models of development and aging

**DOI:** 10.1186/s13104-023-06673-9

**Published:** 2024-01-11

**Authors:** Phyo W. Win, Julia Nguyen, Amanda L. Morin, Charles E. Newcomb, Shiva M. Singh, Noha Gomaa, Christina A. Castellani

**Affiliations:** 1 Department of Pathology and Laboratory Medicine, Schulich School of Medicine and Dentistry, Western University, London, Canada; 2 Department of Biology, Western University, London, Canada; 3 Department of Genetic Medicine, McKusick-Nathans Institute, Johns Hopkins University School of Medicine, Baltimore, USA; 4 Oral Medicine, Schulich School of Medicine and Dentistry, Western University, London, Canada; 5 Department of Epidemiology and Biostatistics, Schulich School of Medicine and Dentistry, Western University, London, Canada; 6 Children’s Health Research Institute, Lawson Research Institute, London, Canada

**Keywords:** Mitochondrial DNA copy number, Epigenetic age, qPCR, Mitochondrial DNA, DNA methylation

## Abstract

**Objective:**

Mitochondrial dysfunction and nuclear epigenetic alterations, two hallmarks of aging, are associated with aberrant development and complex disease risk. Here, we report a method for the simultaneous assessment of mitochondrial DNA copy number (mtDNA-CN) and DNA methylation age (DNAm age) from the same DNA extraction using quantitative polymerase chain reaction (qPCR) and array data, respectively.

**Result:**

We present methods for the concurrent estimation of mtDNA-CN and DNAm age from the same DNA samples. This includes qPCR to estimate mtDNA-CN, representing the number of circular mitochondrial genomes in a cell, and DNA methylation microarray data to estimate the epigenetic age of an individual. Further, we provide a method for the combination of these metrics into a shared metric termed ‘mtEpiAge’. This approach provides a valuable tool for exploring the interplay between mitochondrial dysfunction and nuclear epigenetic alterations, and their associations with disease and aging.

## Introduction

The human lifespan is the result of a complex interplay of genetic, random, and environmental factors, with nine hallmarks of aging shaping its course [1]. Among these, mitochondrial dysfunction and nuclear epigenetic changes have gained prominence due to their variability over the life course. Mitochondrial DNA copy number (mtDNA-CN), a marker of mitochondrial function, varies across cell types, energy reserves, and is associated with mortality, aging, and age-related diseases [2–6]. Environmental factors, including chemicals and drugs, can alter mtDNA-CN, leading to health impacts [7–9]. Epigenetic clocks, like the Horvath epigenetic clock, estimate epigenetic age using age-related CpG sites, revealing insights into aging’s relationship with health and diseases [4, 10–13]. Associations have been established between mtDNA-CN and DNAm Age with a variety of health outcomes, such as cardiovascular disease, cancer, and mortality [2, 14–17]. Therefore, mtDNA-CN and DNAm Age provide two useful metrics for the prediction of disease risk. In this context, we present a comprehensive experimental pipeline for concurrently assessing mtDNA-CN and DNAm Age from the same samples. Our newly proposed metric, mitochondrial-associated DNA methylation age (mtEpiAge), combines these two biomarkers, offering a valuable tool for exploring the interplay of mitochondrial dysfunction and nuclear epigenetic changes on age-related outcomes.

### Materials

All solutions and dilutions were prepared using molecular-grade water. Where additional instructions are not specified, we employed the use of analytical-grade reagents and implemented kits following manufacturer instructions. Reagents were prepared and stored at room temperature unless otherwise noted. For mtDNA-CN estimation via qPCR, we adopted the monochrome assay by Hsieh et al. (2018), which was further optimized by the Arking Lab (Johns Hopkins University School of Medicine) [18]. DNA extraction was performed using the phenol-choloroform-isoamyl alcohol (PCIAA) extraction method. Our protocol was optimized on cell line models, blood, and buccal samples, and can be further adapted to other sample types. For PCIAA DNA extraction, the reagents required were glycogen (20 µg/µL), 3 M Sodium Acetate (pH 5.2), Phenol:choloroform:isoamyl alcohol (25:24:1) (PCIAA) (Invitrogen), 100% and 70% ethanol, and Buffer RLT Plus (Qiagen).

For our qPCR assay, the materials used were Powertrack SYBR Green Master Mix (ThermoFisher Scientific), a MicroAmp Fast Optical Reaction Plate (Applied Biosystems), molecular-grade water, optically clear PCR plate seals (ThermoFisher Scientific), the Qubit BR dsDNA assay kit (Invitrogen), and a Qubit 4 Fluorometer (ThermoFisher Scientific) (or a Nanodrop) for quantification. The Viia7 qPCR thermocycler, (ThermoFisher Scientific) with the provided Viia7 Software version 1.2.2 was used. However, an alternative thermocycler may be used, as required. Lastly, qPCR primers for Albumin (ALB) and D-Loop (DLP) were ordered according to Table 1. Sequences with ‘R’ bases denote degenerate A/G bases (50%/50%). Note that the first 27 base pairs from the 5’ end for the ALB primers have GC-clamps, and the first 12 base pairs from the 5’ end for the DLP primers are non-complementary bases.

**Table 1:** Albumin and mtDNA D-Loop primer sequences. Bold text represents GC clamps; italic text represents non-complementary bases

	Forward Primer Sequence	Reverse Primer Sequence
Albumin	5’ - **CGG CGG CGG GCG GCG CGG GCT GGG CGG** AAA TGC TGC ACA GAA TCC TTG − 3’	5’ - **GCC CGG CCC GCC GCG CCC GTC CCG CCG** GAA AAG CAT GGT CGC CTG TT – 3’
D-Loop	5’ - *ACG CTC GAC ACA* CAG CAC TTA AAC ACA TCT CTG C – 3’	5’ - *GCT CAG GTC ATA C*AG TAT GGG AGT GRG AGG GRA AAA – 3’

For estimating DNAm age, the materials used were the EZ-96 DNA Methylation Kit (Zymo Research) (can be substituted for an equivalent bisulfite conversion kit), a Universal Methylated DNA Standard (Zymo Research), the Infinium MethylationEPIC Kit (Illumina), and the R programming language version 4.2.2 or higher with appropriate dependencies as outlined below.

## Methods

### PCIAA DNA extraction

The recommended DNA extraction method for estimating mtDNA-CN and DNA methylation age is an organic solvent extraction approach using PCIAA. This method has been shown to yield lower variability in mtDNA-CN estimation compared to silica-based column methods [19]. We recommend using the same extracted DNA sample for both mtDNA-CN estimation and EPIC array analysis. The procedure we followed involved the following steps:

- A minimum of 4mLs of cells were collected in a 15mL tube.
- The tube was centrifuged at 2500 x g for 5 minutes and pellet formation was visually confirmed.
- The supernatant was removed from the 15mL tube.
- The pellet was lysed with 350µL of RLT Plus buffer and transferred into a 1.5mL tube.
- 350µL of PCIAA was added to the sample, and vortexed thoroughly for approximately 10s or until the solution turned milky white.
- The tube was centrifuged at room temperature for 15 minutes at 14,000 x g.
- A total of 200–300µL of the top upper aqueous phase was carefully skimmed and the layer was transferred to a new 1.5mL tube. 50–100µL of the upper aqueous phase was removed to ensure only the aqueous phase was taken.
- For ethanol precipitation, the following steps were performed in sequential order:
  - 1µL glycogen was added to the total skimmed volume in the 1.5mL tube.
  - The pre-determined amount of sodium acetate was added in µL, calculated by multiplying 0.1 by the total µL volume of sample collected from the upper aqueous phase.
  - We then multiplied 2.5 by the total sum volume of the sample plus sodium acetate to determine the total amount of 100% ethanol that was added to the tube.
- The combined solution was vortexed and placed at -20 ^o^C overnight to precipitate the DNA. Should you want to proceed with the protocol immediately, the tube can be frozen at -80 ^o^C for 1 hour rather than overnight.
- Samples were placed on ice and centrifuged at 4 ^o^C for 15 minutes at 16,000 x g to pellet the DNA. Pellet formation was visually confirmed.
- The supernatant was carefully removed without disturbing the DNA pellet.
- 200µL of 70% ethanol was added and the DNA pellet was washed by gently pipetting the solution up and down.
- The samples were centrifuged at 4 ^o^C for 10 minutes at 14,000 x g and any remaining ethanol and liquid were removed.
- With an open 1.5mL tube, the DNA pellet was air dried for 15 min at the bench. A Kimwipe was used to cover the tube to prevent any dust/contamination from entering the tube.
- The DNA pellet was re-suspended in 150µL of sterile water by pipetting the solution up and down vigorously until the DNA pellet detached and broke apart. The tube was vortexed thoroughly for 20s, ensuring fully resuspended DNA.
- The tube was centrifuged briefly to collect the sample and was placed back on ice.
- The extracted DNA was stored at -20 ^o^C.
- Depending on the DNA quality (as measured by nanodrop), DNA was further purified with an ethanol precipitation clean-up by reprecipitation with the same steps as mentioned previously. When a second ethanol wash was performed, volume of glycogen, sodium acetate, and 100% ethanol was adjusted according to the volume used to resuspend the DNA as per the protocol above.

### Monochrome multiplex qPCR assay for mtDNA-CN estimation

For qPCR reactions, 20ng of DNA was used per sample, which can be reduced to 10ng if DNA is limited. Samples were run in technical triplicates for all runs. Working stocks of primers were prepared by diluting parent stocks to 40µM in water and stored at -20 °C. DNA samples were assessed for quantity and quality, ensuring 260/280 and 260/230 ratios of ~ 1.8 and ~ 2.0-2.2, respectively. DNA quantification was done using a Qubit Fluorometer or a Nanodrop, with the former providing a higher accuracy for quantification. DNA was diluted to 10 ng/µL concentration. qPCR primers and SYBR green master mix were thawed on ice and the master mix was protected from light. qPCR master mix was created using the values in Table 2, with appropriate adjustment of reagent volumes for the total number of samples and replicates performed. The mix was vortexed and centrifuged. 8µL of master mix and 2µL of diluted DNA were combined in each sample well and triplicate samples were randomized on the plate to reduce technical effects. An optical clear seal was used to seal the plate and the plate was vortexed and centrifuged. For qPCR on the Viia7 machine (or similar), an experiment setup with requisite settings was created. Data were captured at 74 °C and 88 °C for DLP and ALB, respectively. The plate was loaded and the run was started.

**Table 2:** Required reagents for qPCR master mix per sample

Reagent	1x Reaction Volume (µL)
Water	2.0
ALB-Forward Primer (40µM)	0.25
ALB-Reverse Primer (40µM)	0.25
DLP-Forward Primer (40µM)	0.25
DLP-Reverse Primer (40µM)	0.25
Powertrack SYBR Green Master Mix	5.0
**Total Volume**	8.0

mtDNA-CN estimates were derived from Cycle Threshold (Ct) values. DLP Ct values ranged within 17–20 Cts, while ALB Ct values fell within 25–30 Cts. Ct data was exported as a CSV file, DLP Ct readings > 26 were filtered out. For samples with replicates, outliers > 3 SD from the mean Ct were removed. Delta Ct was calculated as ALB Ct – DLP Ct and samples with delta Ct < 7 were filtered out. Pipetting order effects were also addressed at this stage. Consistency of ALB Ct values across samples was reviewed to reduce any nuclear DNA variation. Final delta Ct values were visualized using ggplot2.

### Estimation of DNA methylation age (DNAm age)

Bisulfite treatment of purified DNA was performed using the EZ-96 DNA Methylation Kit following the manufacturer’s protocol (Zymo Research), with a minimum of 1 µg of genomic DNA used as input. Bisulfite conversion efficiency was verified by running PCR amplification with Zymo Research’s Universal Methylated Human DNA Standard and Control Primers (Catalog number D5011). DNA was hybridized to the Illumina Infinium MethylationEPIC BeadChip kit and DNAm profiles were determined across 850,000 CpG sites in the human genome. For quality control of methylation data, we used the R packages minfi, ChAMP, and RnBeads. The packages require that you read in raw methylation .idat files along with phenotype data. Preprocessing of methylation data was done by removing poor-quality samples (detection p-value cutoff of 0.01), common SNPs, and cross-reactive probes. The Pidsley et al. list of cross-reactive probes was used [20]. Appropriate data normalization using the minfi package [21] was performed and methylation beta-values were extracted. For DNAm Age (Horvath Clock), required files and dependencies were installed as per Horvath’s guide (https://horvath.genetics.ucla.edu/html/dnamage/). The annotation file and coefficients for the 353 methylation age CpGs were loaded [22]. Additionally, the preprocessed methylation beta-values from above were filtered for CpGs of interest that are used to estimate DNAm age [23]. After applying the code provided from Horvath (2013) on the normalized values, final DNAm age values were extracted from the DNAmAge column of the output.csv file.

### Estimation of mtDNA-CN associated DNAm age (mtEpiAge)

To generate mtEpiAge, the stratified quantiles of each individual metric were first calculated (mtDNA-CN and DNAm age). The average of the scaled estimates for both mtDNA-CN and DNAm age of each quantile was used to generate a new mtDNA-CN associated DNAm age estimate by taking the difference between each scaled estimate and their respective quantile average value. This was done with the following steps: DNAm Age and mtDNA-CN estimates from previous steps were loaded into the current R environment. Values were merged by sample name into the same dataframe. DNAm Age values and mtDNA-CN estimates were scaled to a range of 0-100 for ease of data manipulation. Resulting values were saved to columns named “Age_scaled” and “CN_scaled” respectively. Values from the “Age_scaled” column were annotated into four stratified quantiles using the quantile function and sorted into bins representing each quantile using the cut function. Similarly, values from the “CN_scaled” column were annotated into four stratified quantiles. The mean of each of the four quantiles was computed for both the “Age_scaled” values and the “CN_scaled” values. A new column labeled “Age_QMean” to store the mean value of the respective quantile for each value in “Age_scaled” was created. Additionally, a new column labeled “CN_QMean” that stores the mean value of the respective quantile for each value in “CN_scaled” was also created. For each row in the dataframe, the average of “CN_QMean” and “Age_QMean” was computed, followed by the average of “Age_scaled” and “CN_scaled”. The absolute difference between the two computed averages was reported as the mtDNA-CN associated DNAm age estimate (mtEpiAge). The mtEpiAge provides a useful metric to further explore the interplay between mitochondrial dysfunction via mtDNA-CN and associated nuclear epigenetic age-related variation through DNAm age.

### Future directions

Associations between disease risk and mtDNA-CN [2, 7, 14–16, 24] as well as DNAm age [10–13, 25] have been identified for a variety of health outcomes, including cardiovascular disease, cancer, liver disease, and all-cause mortality. These associations demonstrate the potential utility of an aggregate measure of mtDNA-CN and DNAm age. However, most studies have reported only independent associations with disease for mtDNA-CN and DNAm age. Given the advent of omic technologies, studies are emerging with matched nuclear DNAm and mtDNA-CN data from the sample samples and these studies have identified significant associations between mtDNA-CN, DNA methylation, and disease [24, 26–28]. Therefore, to leverage these results, we propose the simultaneous assessment of mtDNA-CN and DNAm age using mtEpiAge towards increased predictive power for disease risk. Integration of additional molecular markers with mtEpiAge would be expected to further refine the applicability of mtEpiAge as a tool in determining the relationship between mitochondrial function, epigenetic aging, and health outcomes.

### Limitations

Variations in DNA extraction efficiency can impact DNAm Age estimates, potentially leading to measurement errors. mtDNA estimation can also be affected by multiple freeze-thaw cycles and choice of DNA extraction method, as demonstrated by lower variability in mtDNA estimation with PCIAA techniques compared to traditional silica-based column selection methods [19, 29].

## Data Availability

NA.

## Abbreviations

ALB: Albumin
DLP: D-loop
^o^C: Degree Celsius
CN: Copy number
Ct: Cycle threshold
DNA: Deoxyribose nucleic acid
DNAm: DNA methylation
dsDNA: Double stranded DNA
mtDNA: Mitochondrial DNA
mtEpiAge: MtDNA-CN associated DNAm age estimate
µL: Microliter
mL: Milliliter
M: Molar
µM: Micromolar
µg: Micrograms
ng: Nanograms
PCIAA: Phenol:choloroform:isoamyl alcohol
PCR: Polymerase chain reaction
qPCR: Quantitative PCR
s: Seconds
SNP: Single nucleotide polymorphism
