# Correction: Simultaneous assessment of mitochondrial DNA copy number and nuclear epigenetic age towards predictive models of development and aging

**DOI:** 10.1186/s13104-025-07260-w

**Published:** 2025-04-27

**Authors:** Phyo W. Win, Julia Nguyen, Amanda L. Morin, Charles E. Newcomb, Shiva M. Singh, Noha Gomaa, Christina A. Castellani

**Affiliations:** 1 Department of Pathology and Laboratory Medicine, Schulich School of Medicine and Dentistry, Western University, London, Canada; 2 Department of Biology, Western University, London, Canada; 3 Department of Genetic Medicine, McKusick-Nathans Institute, Johns Hopkins University School of Medicine, Baltimore, USA; 4 Oral Medicine, Schulich School of Medicine and Dentistry, Western University, London, Canada; 5 Department of Epidemiology and Biostatistics, Schulich School of Medicine and Dentistry, Western University, London, Canada; 6 Children’s Health Research Institute, Lawson Research Institute, London, Canada

**Correction: BMC Research Notes (2024) 17:21**

10.1186/s13104-023-06673-9

Following the publication of the original article [1] we were informed of a typographical error in author Julia Nguyen’s name.

The author’s family name “Nguyen” was incorrectly presented as “Nyugen”.

The correct name is shown in the author list and reference of this Correction. The original article has been corrected.

